# Healthcare Professionals’ Perspectives on Post-Diagnostic Care for People with Vascular Cognitive Impairment: When Help Is Needed in a “No-Man’s Land”

**DOI:** 10.3233/JAD-240526

**Published:** 2024-09-24

**Authors:** Sara A.J. van de Schraaf, Hanneke F.M. Rhodius-Meester, Lindsey M. Rijnsent, Meyrina D. Natawidjaja, Esther van den Berg, Frank J. Wolters, J.M. Anne Visser-Meily, Geert Jan Biessels, Marjolein de Vugt, Majon Muller, Cees M.P.M. Hertogh, Eefje M. Sizoo

**Affiliations:** a Medicine for Older People, Amsterdam UMC, Location VUmc, Amsterdam, The Netherlands; b Internal Medicine, Geriatric Medicine Section, Amsterdam UMC, Location VUmc, Amsterdam, The Netherlands; c Amsterdam Public Health, Aging & Later Life, Amsterdam, The Netherlands; d Neurology, Alzheimer Center Amsterdam, Amsterdam UMC, Location VUmc, Amsterdam, The Netherlands; e Amsterdam Neuroscience, Neurodegeneration, Amsterdam, The Netherlands; f Department of Geriatric Medicine, Oslo University Hospital, Oslo, Norway; g Department of Neurology and Alzheimer Center, Erasmus MC University Medical Center, Rotterdam, The Netherlands; h Department of Radiology & Nuclear Medicine, Erasmus MC University Medical Center, Rotterdam, The Netherlands; i Department of Epidemiology, Erasmus MC University Medical Centre, Rotterdam, The Netherlands; j Department of Rehabilitation, Physical Therapy Science & Sports, University Medical Centre Utrecht, Utrecht, The Netherlands; k UMC Utrecht Brain Center, University Medical Center Utrecht, Utrecht, The Netherlands; l Department of Neurology and Neurosurgery, University Medical Center Utrecht, Utrecht, The Netherlands; m School for Mental Health and Neuroscience, Alzheimer Center Limburg, Maastricht University, Maastricht, The Netherlands; n Amsterdam Cardiovascular Sciences, Atherosclerosis & Ischemic Syndromes, Amsterdam, The Netherlands

**Keywords:** Aftercare, Alzheimer’s disease, cerebrovascular diseases, patient care management, post-stroke cognitive impairment, rehabilitation, vascular cognitive impairment, vascular dementia

## Abstract

**Background:**

Post-diagnostic care for people with vascular cognitive impairment (VCI) typically involves multiple professions and disjointed care pathways not specifically designed to aid VCI needs.

**Objective:**

Exploring perspectives of healthcare professionals on post-diagnostic care for people with VCI.

**Methods:**

We conducted a qualitative focus group study. We used purposive sampling to include healthcare professionals in different compositions of primary and secondary care professionals per focus group. Thematic saturation was reached after seven focus groups. Transcripts were iteratively coded and analyzed using inductive thematic analysis.

**Results:**

Forty participants were included in seven focus groups (4–8 participants). Results showed knowledge and awareness of VCI as prerequisites for adequate post-diagnostic care, and for pre-diagnostic detection of people with VCI (theme 1). In light of perceived lack of differentiation between cognitive disorders, participants shared specific advice regarding post-diagnostic care for people with VCI and informal caregivers (theme 2). Participants thought current care for VCI was fragmented and recommended further integration of care and collaboration across settings (theme 3).

**Conclusions:**

People with VCI and their caregivers risk getting stuck in a “no man’s land” between post-diagnostic care pathways; challenges lie in acknowledgement of VCI and associated symptoms, and alignment between healthcare professionals. Education about the symptoms and consequences of VCI, to healthcare professionals, people with VCI and caregivers, may increase awareness of VCI and thereby better target care. Specific attention for symptoms common in VCI could further tailor care and reduce caregiver burden. Integration could be enhanced by combining expertise of dementia and stroke/rehabilitation pathways.

## INTRODUCTION

Cerebrovascular pathology is the second most common contributor to dementia,^1–4^ after Alzheimer’s disease. Vascular cognitive impairment (VCI) is an umbrella-term covering the full spectrum of cognitive impairment associated with cerebrovascular pathology, from mild cognitive changes to (vascular) dementia. People with VCI can present in healthcare in several ways. For example, with acute onset decline due to stroke, or with gradual cognitive decline due to cerebral small vessel disease.^5^ Therefore, a wide variety of healthcare professionals in different post-diagnostic care pathways is involved with people with VCI. These professionals work with their own guidelines in separate workflows, which might negatively affect cohesion and increase the risk of fragmentation. Earlier studies have noted that fragmentation can lead to diminished quality and efficiency of care, and creates challenges in navigating the care landscape for the patient and their caregiver.^6,7^

Internationally, post-diagnostic care pathways supporting people with VCI include stroke and rehabilitation care and dementia care.^8,9^ Patient and professional perspectives on these two pathways have been described in the literature. In stroke and rehabilitation care, fragmented workflows, variable quality of (transmural) communication and lack of continuity in care have been described.^10–14^ These challenges were particularly described concerning cognitive and emotional symptoms after stroke, even though the majority people of suffering from stroke exhibit cognitive impairment, even after successful recovery.^15^ Healthcare professionals working in dementia care wish to move towards more coordinated and integrated post-diagnostic care, for example by a continuous and single point of contact or more collaboration between different professionals.^16–20^ Yet, literature about the appropriateness of these care pathways for people with VCI specifically is lacking. People with VCI and their caregivers may be at risk to fall through the cracks of the different care pathways not specifically aiding their needs.

Previously, we studied post-diagnostic care needs of people with VCI and their caregivers.^21^ One of the main outcomes was a need for information about VCI, particularly regarding common symptoms and regarding the difference between VCI and other cognitive disorders such as dementia. People with VCI and caregivers tended to equate cognitive impairment to memory loss or Alzheimer’s disease. Furthermore, people with VCI and caregivers expressed the need to be supported by professionals in managing symptoms common in VCI, such as apathy. In order to relay these findings to clinical care, it is important to include perspectives of healthcare professionals, as views on current management of VCI among various types of healthcare professionals are unknown.

Thus, the perspective of healthcare professionals is needed to gain more insight in professional and organizational aspects of post-diagnostic care for people with VCI. While previous studies have focused on stroke or dementia pathways separately, we adopt an integrated approach, including healthcare professionals across care pathways in primary and secondary care in different group compositions. In this qualitative study, we discuss the perspectives of these healthcare professionals on post-diagnostic care for people with VCI and their caregivers.

## METHODS

This study was conducted and reported in accordance with the COREQ criteria for qualitative research (Supplementary Table 1).^22^

### Design

We chose a focus group design; focus groups capitalize on group interactions, encouraging participants to clarify their statements and explore the perspectives and opinions of other participants.^23^ Through interactive discussion, we aimed to achieve better understanding of experiences and challenges faced by various professionals.

Focus groups were organized in different urban and rural regions across the Netherlands. We varied focus group composition, to ensure optimal variation in healthcare professionals and discussed topics in different focus groups. We organized focus groups with professionals working in primary care (2 groups) and secondary healthcare settings (3 groups), and also organized focus groups including professionals from both settings (2 groups).

### Participants and sampling

Eligible participants included healthcare professionals working with people with cognitive impairment, either in stroke or dementia care pathways. We approached physicians (including geriatricians, neurologists, general practitioners and elderly care physicians),^24^ nurses, psychologists, allied health professionals, and case managers in dementia care.

The sampling procedure was a combination of convenience and purposive sampling. Participants were approached within the professional networks of the authors through e-mail and phone. We also asked potential participants to consult their network for interested colleagues. We used purposive sampling (intentionally approaching certain potential participants), in order to achieve sufficient variety of information-rich cases.^25^ We sampled on variation in age, sex, profession, setting, and years of professional experience with people with cognitive impairment: e.g. included younger participants if these were lacking in the sample, and so forth. The present work is in accordance with the Helsinki Declaration of 1975. The Medical Ethics Committee of the Amsterdam UMC granted study approval (METC 2020.0746) and all participants provided informed consent.

### Procedure

Focus groups were guided by ES (experienced senior qualitative researcher, moderated focus groups 1–6) and SvdS (trained and experienced in qualitative research, moderated focus group 7) between July 2022 and March 2023. Field notes were taken during each focus group session by the observer (SvdS or ES), mainly focusing on non-verbal communication. After each focus group session, we debriefed according to a checklist and noted remarkable observations and key points from the discussion. All focus groups were audiotaped and transcribed verbatim. Identifiable information was pseudonymized during transcription.

The research team developed a topic-list before data-collection. The topic list included open-ended questions focusing on the participants’ views on (1) current (stroke and dementia) post-diagnostic care (2) possible caveats in post-diagnostic care for people with VCI and caregivers and (3) organizational issues. The topic list was revised several times during data collection, primarily to accommodate the different participants or settings (Supplementary Table 2).

During each focus group, we defined the VCI group as “people with cognitive impairment, ranging from mild cognitive impairment to dementia level, where vascular etiology is the most likely and prominent cause”. We chose this definition to make sure participants focused on VCI and not mixed etiologies, and on people with objective cognitive impairment who are eligible for receiving professional care.

Data-collection ended at thematic saturation, meaning additional information and data did not contribute to new (sub-)themes.^26^ At the seventh focus group, no novel topics were discussed that led to different (sub-) themes; therefore, we concluded that saturation was reached.

After data-collection and analysis, we organized two opportunities for our participants to attend a presentation of the outcomes and proposed themes. Return of transcripts or individual data to the participants was not deemed appropriate, as this would imply that individual participants could reconstruct the narrative.^27^ Participants were asked to comment on whether the proposed themes reflected their understanding of the topic: an interpretive stance on member checking (assessing the trustworthiness of the analysis of the data). Our participants agreed on the proposed themes, while providing feedback on what findings seemed particularly important or striking to them. This feedback assisted with structuring of the themes and deciding which aspects to highlight.

### Data analysis

The data was coded and analyzed using the inductive thematic analysis approach by Braun and Clarke^28^ in MAXQDA 2022.

The first step involved obtaining an in-depth understanding of the data by reading and re-reading the transcripts. At least two researchers (SvdS and LR or MN, trained by SvdS and ES) independently coded each transcript (step 2), identifying open codes derived from the data (inductive coding). Codes were arranged in superordinate and subordinate categories and themes using an axial and iterative coding process (Step 3; coding cloud in Supplementary Figure 1). Themes were discussed and refined in project meetings until consensus was reached (step 4), after which the themes were named (step 5). All authors approved the final themes, after which the report was written (step 6).

## RESULTS

### Participants

Forty healthcare professionals were included over seven focus groups (see Table 1 for distribution of professions over focus groups Table 1). There were 4–8 participants per focus group. All focus groups lasted approximately 90 min. The majority of participants were women (78%), and there was a wide variety in age (29–67 years) and years of relevant experience (3–37 years).

**Table 1:** Participant distribution across the focus groups

		FG1	FG2	FG3	FG4	FG5	FG6	FG7
		(*n* = 6)	(*n* = 5)	(*n* = 4)	(*n* = 4)	(*n* = 7)	(*n* = 6)	(*n* = 8)
		Secondary care	Secondary care	Secondary care	Primary care	Mixed	Mixed	Primary care
Participant type	N
Elderly care physician	6	1			2		1	2
Geriatrician	4		1	1			2
Internist-Geriatrician	3	1	1			1
Neurologist	3	2					1
General practitioner	3					2		1
Case manager in dementia care	5				2	1	1	1
Practice nurse	3					2		1
Ambulatory nurse	1							1
Outpatient-clinic nurse	3	2				1
Psychologist	4			2			1	1
Occupational therapist	2		1					1
Social worker	3		2	1

FG = Focus group.

### Themes

**Fig. 1:**
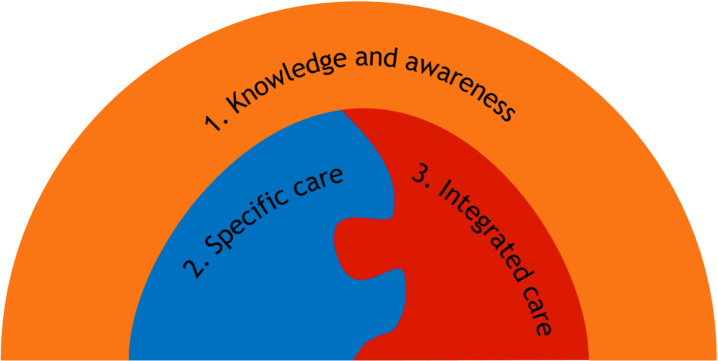
Three major themes of healthcare professionals’ perspectives on post-diagnostic care for people with vascular cognitive impairment and their caregivers.

Three major themes were identified in the responses of participants: (theme 1) “With Knowledge and awareness, the rest will come”, (theme 2) Specific care: “A population with different needs” and (theme 3) Integrated care: “Breaking down the barriers” (Fig. 1).

#### Theme 1. “With Knowledge and awareness, the rest will come”

We classified this theme as an overarching theme, meaning the participants argued that knowledge and awareness about VCI in healthcare professionals and the general public were a prerequisite for providing appropriate post-diagnostic care (subtheme 1.1), but also for detection of people with VCI at a pre-diagnostic stage (subtheme 1.2).

*Subtheme 1.1. Knowledge in post-diagnostic care: “Understanding the behavior”* In general, participants remarked that knowledge about VCI varied greatly between professionals. They were concerned about the level of knowledge in primary care, where the majority of post-diagnostic care and support takes place, but is spread out between many providers. They agreed that within primary care, there is basic knowledge of dementia and stroke, but not about VCI specifically. Participants stated that more knowledge about the specific causes, symptoms and consequences for daily life was needed in post-diagnostic care networks. With sufficient knowledge of VCI, awareness of the condition should increase. This could be the gateway to better-targeted care:

“*Knowing about the issues. How important that is, that should really be in the foreground. And I think if you have that awareness, the rest will come automatically.*” (FG6, geriatrician)

Insufficient knowledge of VCI had several consequences according to the participants. Firstly, participants noted that it might be hard to distinguish certain symptoms, such as decreased processing speed or cognitive decline in the years after stroke, from normal aging. This might lead to misinterpretation of symptoms and behavior. Secondly, the participants noted that insufficient knowledge of VCI could also lead to failure to acknowledge the pattern of symptoms as a cognitive disorder or dementia, especially if the person has good memory or disease awareness. Consequently, this could lead to overestimation of functioning and capacities of people with VCI:

“ *[Sometimes] I think: these people are still in far too good a state for day care. [*…*] They end up in a welcoming environment, a new family. Then I often think: Oh, I overestimated them, or they may have been walking a fine line for a while.*” (FG1, elderly care physician)

According to the participants, professional knowledge and awareness of VCI are also needed to educate people with VCI, caregivers and the public. In the general population, VCI is not as well-known as for instance Alzheimer’s disease, and therefore its consequences are not as well understood. The aforementioned overestimation was also a problem for people with VCI and informal caregivers. Participants noticed it was hard for them to recognize the symptoms they experienced as something similar to dementia:

“*All kinds of other symptoms, but not necessarily memory problems. And you can encounter quite a bit of resistance if you start talking about vascular dementia:* ‘*Yes, but my father or mother doesn*’*t have dementia. Because it*’*s really not that bad*’.” (FG6, geriatrician)

More awareness of the consequences of VCI would facilitate more understanding and acknowledgement in people with VCI and informal caregivers. In order to increase knowledge in the people affected, participants noted that one of the most important tasks of the healthcare professional is to provide targeted psycho-education. They elaborated that psycho-education can aid acceptance and coping in the person with VCI and their caregiver:

“*It helps the relative if they have a better understanding of the patient*’*s behavior, right. That*’*s important, if you can explain to people why the patient is slow, why they don*’*t take any initiative and why they sit around all day.*” *(FG1, neurologist)*

*Subtheme 1.2. Pre-diagnostic detection of VCI: “They don’t recognize it”* Although participants were instructed to focus on post-diagnostic care, they highlighted that limited knowledge and awareness of the clinical presentation of VCI was a barrier to pre-diagnostic detection of people with VCI, especially within primary care or after stroke. Due to insufficient recognition of the symptoms in primary care, people with VCI are at risk of not receiving a timely or correct diagnosis, and therefore appropriate (post-diagnostic) care cannot be initiated:

“*Many GPs are indeed not that well informed about the clinical picture, or they don*’*t recognize it. So people can continue with symptoms for a long time and not know where they can [go for help].*” (FG2, social worker)

Participants noted that one of the challenges of recognizing VCI in primary care was the gradual decline of cognition that could remain unnoticed if not recognized appropriately. Some thought that it would be easier to recognize VCI in people who had a stroke, due to the acute onset. However, other participants debated this. They highlighted that the emphasis in stroke aftercare and rehabilitation is on functional recovery, and not cognition. Therefore, cognitive decline is not always recognized and therefore not properly assessed or diagnosed as VCI:

“*So that is often followed by a rehabilitation process, but that really focuses on the basics. And I regularly see people in neurology [outpatient clinic] who basically come back three years later and say,* ‘*Well, I*’*m actually suffering a lot from those cognitive complaints or mood problems*’.” (FG3, psychologist)

#### Theme 2. Specific care: “A population with different needs.”

Within this theme, healthcare professionals shared specific advice regarding post-diagnostic care for people with VCI. Participants perceived a lack of differentiation of the VCI group (subtheme 2.1). They proposed a specific approach for people with VCI, focusing on common symptoms (subtheme 2.2) and highlighted that caregivers face specific challenges (subtheme 2.3).

*Subtheme 2.1. Is specific care needed? “Currently no differentiation”* At the start of some focus groups, a discussion emerged between the participants about whether specific care or recommendations are needed for VCI, as it has a heterogeneous clinical presentation. Some participants stated that they did not particularly focus on different causes of cognitive impairment when providing care, but noted that their main focus is providing person- and needs-centered care. Although all types of professionals agreed to this to some extent, these statements were particularly provided by generalists in primary care such as case managers or general practitioners:

“*I have to be honest that I haven*’*t always thought: What was it [the diagnosis of this person] exactly? Vascular or Alzheimer*’*s? [*…*] What I do look at is: who were you and how do you see your future?*” (FG5, case manager)

On the other hand, the participants agreed that more knowledge and awareness of VCI, by differentiating it from other cognitive disorders, would be beneficial for healthcare professionals (Theme 1). Participants explained that adequate knowledge of VCI can aid a professional by providing benchmarks against which an individual’s behavior can be judged. In this manner, adequate knowledge of VCI can guide healthcare professionals to provide targeted post-diagnostic care and give adequate psycho-education:

“*[That the] VCI group is very much a different patient population with different needs. [*…*] That there comes a point when it certainly helps to set out some markers so that people [get] information that is directly related to the real-life situation.*” *(FG2, internist-geriatrician)*

Furthermore, participants perceived that a lack of differentiation with other causes of cognitive impairment could be disadvantageous for people with VCI. In some cases, participants felt that ‘regular’ dementia or stroke care would not align with the needs of a person with VCI. They provided several examples of situations in which this could be the case, such as people with relatively mild cognitive disorder (“not yet dementia”), people with fluctuations in functioning or people with a subcortical pattern of cognitive deficits (impaired processing speed, no memory deficits):

“*The fact that it*’*s often very mild or the memory isn*’*t [affected] to any great extent. [*…*] People don*’*t always feel particularly comfortable on a ward or in a day center.*” *(FG1, elderly care physician)*

*Subtheme 2.2. Care for the person with VCI: “More dynamic and tailored care”* This subtheme covers the specific advice healthcare professionals had for post-diagnostic care in people with VCI. Several different symptoms and characteristics of VCI require special attention according to the participants.

Participants working in stroke care pathways emphasized that people with VCI often need long-term care and guidance, compared to other patient groups they encounter in their work. They described the VCI group as a group with permanent cognitive changes, for whom rehabilitation should be targeted to coping with cognitive deficits, rather than recovery of function. Therefore, the emphasis in VCI rehabilitation should be on chronic care and guidance:

“*That you soon have to look in terms of strategies [in treatment]. That*’*s different with someone [*…*] whom you*’*re expecting in principle to recover. So lengthy, long-term problems that you*’*re not going to resolve just like that.*” (FG2, occupational therapist)

Participants proposed more flexibility in the approach of people with VCI. They stated that VCI is a heterogenous condition with a varied clinical presentation, therefore there is no one-size-fits-all approach. On top of that, they felt VCI often came with fluctuations in functioning (good days and bad days). For these situations, a flexible and dynamic approach with varying amounts of autonomy could also be beneficial:

“*For the care that is offered to be dynamic and therefore responsive, right. So if a patient is doing well, they could be given more independence and need less support and be more autonomous, and it*’*s OK for that to fluctuate.*”(FG1, internist-geriatrician)

Even though participants described VCI as heterogenous, there was overall agreement on some common characteristics symptoms. These symptoms included fluctuations in functioning (see quote above), executive dysfunction, decreased processing speed and apathy. Participants provided specific advice for these characteristics symptoms, requiring a particular approach from healthcare professionals:

“*Be somewhat more directive. Of course, you want to keep things on an equal footing by letting the person take their own decisions or make their own choices. But if someone is really apathetic, that doesn*’*t work well.*” *(FG3, psychologist)*

*Subtheme 2.3. Attention for caregivers: “They have huge care needs”* Participants repeated this statement across focus groups: informal caregivers of people with VCI need special attention. They stated that informal caregivers of people with VCI often have high caregiver burden. One of the reasons participants gave for high caregiver burden was the lack of knowledge and attention for VCI in the media, or even by healthcare professionals (Theme 1). Participants noticed that caregivers of people with VCI often wait long before consulting a professional, as they do not recognize the symptoms as VCI or dementia. In addition, they often do not get acknowledgement from their social environment, who can perceive the person with VCI as functioning quite well. After diagnosis, there are some symptoms that particularly affect caregiver burden. For example, participants highlighted that the non-linear and unpredictable disease course of VCI can cause significant insecurity in caregivers about the prognosis:

“*In vascular dementia, right, it often takes a really long time before someone starts to deteriorate severely. [*…*] And it goes up and down. Then I notice the partner can find that difficult. That kind of no-man*’*s- land. Help is indeed necessary then.*” (FG4, elderly care physician)

Participants noted that apathy was especially burdensome for caregivers, more so than the cognitive consequences of VCI. Participants posed that informal caregivers often do not recognize apathy as something that can be the consequence of a cognitive disorder, and will become frustrated when their attempts to activate the person do not succeed.

“*I often find people with vascular dementia don*’*t really have care needs. It*’*s much more likely for the people around them to have huge needs. Especially due to that apathy, people are often somewhat listless. [*…*] And that is often draining for the people around them.*” (FG5, nurse)

#### Theme 3. Integrated care: “Breaking down the barriers.”

In this theme, the participants described their preferred organization of post-diagnostic care for people with VCI. They felt current care was highly fragmented (subtheme 3.1). Therefore, besides advocating for more differentiation and specialization (theme 2), participants wanted more integration of different settings, professionals and care pathways (subtheme 3.2).

*Subtheme 3.1. Fragmented care: “Very fragmented on their islands”*

“*The interdisciplinary cooperation, purely on the medical side, could and should be better. [*…*] I find it really quite fragmented. Geriatrics is an island, neurology is an island.*” (FG6, geriatrician)

Participants perceived post-diagnostic care pathways as fragmented. In general, within primary care or secondary care, professionals report some degree of collaboration, but participants stated that this varied greatly. Especially in secondary care, collaboration was often constrained mostly to their specific department. Transmural collaboration and collaboration between stroke and dementia pathways was perceived as even more limited. Participants felt this hindered their ability to assume a coordinating role, as they could not grasp the patient’s network. Participants stated that fragmentation could particularly be a problem for people with VCI, who are known across a multitude of pathways and departments due to the high prevalence of comorbidities.

Participants reported several consequences of fragmentation. First, limited knowledge of the other network can be inefficient due to duplication of effort. Second, it might lead to different professionals providing contradictory information. This leads to unnecessary workload for professionals and confusion for people with VCI and caregivers:

“*Then the GP sends all the family caregivers to that welfare organization and they get all this advice there. Then I come with my advice. I think we should also make sure we don*’*t end up duplicating things. That is obviously a waste of time and money.*” (FG6, case manager)

Participants also commented that they felt follow-up of cognitive consequences of stroke was too short-term: follow-up generally ends after a few months and afterwards people with VCI generally do not get long-term assistance. The cognitive follow-up is usually not handed off to another professional, such as the general practitioner. This leaves the responsibility of seeking further care and support to the person with VCI and the caregiver:

“*[Patients end up at a] CVA aftercare outpatient clinic, but that*’*s usually quite soon after the cerebral infarction. So that*’*s usually after only about four weeks. And if people start rehabilitation, then they might be seen or get a phone call after three months, at least that*’*s the case here. [*…*] But I think that*’*s often too soon to, as you say, to really note the invisible effects in the home situation at that stage.*” *(FG6, neurologist)*

*Subtheme 3.2. Collaboration & Communication: “Coming together is worthwhile”* Participants called for more integration: transmural, between different professions within primary or secondary care and between different care pathways. Some participants had more experience than others with transmural and trans-professional collaboration. They emphasized that close collaboration would become increasingly important with the ageing population in mind. According to the participants, the collaboration between specialist (in secondary care) and generalist (in primary care) could particularly be improved. Medical specialists wished to stay involved longer, by supporting primary care professionals managing long-term post-diagnostic care. However, they were not sure if this was desired by primary care:

“*I think it would be a nice question for primary care providers: what do you need from the hospital to keep the [primary] care going? And for example whether*… *Well, how would they feel if someone in the post-diagnostic stage were to see a neurologist or geriatrician, say, twice a year? Just to discuss ideas with one another. Would that help?*” *(FG3, geriatrician)*

Participants recognized that further integration of care could be complex, and raised concerns on the expected costs and feasibility. Participants proposed a warm handoff as a relatively efficient, but very important, way to increase collaboration and coordination. They noted that a warm hand-off could be through phone contact, but preferably in person if feasible (in a multidisciplinary meeting). In this way, prior recommendations could be transferred to the next setting, again avoiding unnecessary duplication of effort.

“*Optimizing the handoff to primary care providers. With the ideas we*’*ve had here about what might help in the home situation and so, right, make that transfer go as well as possible.*” (FG2, social worker)

## DISCUSSION

### Summary of findings

We investigated healthcare professionals’ perspective on post-diagnostic care for people with VCI and their caregivers. Participants’ responses could be categorized in three major themes. We found that knowledge and awareness of VCI were considered prerequisites for adequate post-diagnostic care, as well as for pre-diagnostic detection of people with VCI (theme 1). In addition, healthcare professionals proposed a combination of more specific recommendations (such as directiveness in case of apathy and caregiver support for prognostic uncertainty; theme 2) and more integrated post-diagnostic care (such as increased transmural and trans-professional collaboration across care pathways; theme 3), to optimally meet the needs of people with VCI and their caregivers.

### Knowledge and awareness of VCI

Within the first theme, the need for more knowledge and awareness about VCI for all stakeholders was discussed. In stroke and dementia literature, people and their caregivers express a need for information about the diagnosis and available services.^11,12,19,20,29^ In our study, these findings were complemented by the notion that more knowledge about VCI among healthcare professionals would be helpful to improve care. Primarily, this constituted knowledge about the symptoms and consequences for daily functioning. Participants highlighted VCI characteristics such as fluctuations in functioning, apathy, the possible absence of memory deficits and cognitive and emotional consequences of stroke.^1,30,31^ With this knowledge, post-diagnostic care could be better targeted to a person’s needs (subtheme 1.1).

Participants often made the comparison between VCI and Alzheimer’s disease. Earlier studies report that people believe Alzheimer’s is another word for dementia,^32^ that memory loss is fundamental to dementia,^21^ or that dementia is not associated with cardiovascular disease or risk factors.^33–35^ Consequently, people with cognitive decline that do not align with these stereotypes, such as people with VCI, can be overlooked or their capacities overestimated. This can result in overburdening, neglect or isolation when appropriate care is not initiated timely. More knowledge about VCI and differences with other cognitive disorders in healthcare professionals could decrease these risks. More broadly, restricting the use of the term Alzheimer’s disease to specific pathology, rather than a ‘pars pro toto’ of dementia, may aid in acknowledging other forms and manifestations of cognitive impairment. In addition, healthcare professionals should have sufficient attention and tools for translating their knowledge about VCI to their patients, including family and caregivers, in the form of psycho-education.

Increased knowledge and awareness could also aid pre-diagnostic detection of VCI, particularly after stroke or in primary care (subtheme 1.2). In congruence with the literature, participants commented that there is insufficient awareness of the cognitive consequences of stroke.^11,13^ Other studies have pointed out that primary care professionals, such as general practitioners and practice nurses, often feel they lack knowledge and therefore confidence to detect dementia.^36–38^ One study indicated primary care professionals often do not consider symptoms such loss of initiative and apathy as indicative of possible dementia.^38^ Campaigns to increase professionals’ knowledge and awareness of VCI may be targeted especially to these groups, who collectively deal with most of VCI post-diagnostic care, but see a limited number of people with VCI per professional. In this manner, appropriate post-diagnostic care and support would be more accessible for previously undetected people with VCI.

### Specific care

Within the theme “Specific care,” lack of differentiation between VCI and other cognitive disorders in post-diagnostic care was perceived in two ways. On the one hand, participants did not perceive lack of differentiation as a problem, as person- and needs-centered care was considered the most important.^39^ On the other hand, participants wanted more differentiation based on characteristics of VCI, such as a specific approach to apathy and fluctuations in functioning or considering alternative daycare options. These two viewpoints might not be as contradictory as they seem at first glance. According to participants in our study, providing person-centered care also means being aware of and acting upon (theme 1) how VCI could affect behavior, cognition and caregiver burden in the individual context.

Participants emphasized the importance of attending caregiver burden in VCI. Earlier studies have indicated that caregivers are at risk of high caregiver burden in dementia and stroke populations.^40,41^ Our study adds on to this knowledge by providing examples on why caregiver burden is also a specific concern in VCI, in particular with regard to prognostic uncertainty and apathy.^42–44^ Apathy has been described as being one of the most distressing and relationship-straining behavioral symptoms for informal caregivers.^45^ It is often (mis-)understood as intentional or as a coping strategy.^46,47^ Therefore, supporting informal caregivers and educating them about apathy is important. Caregivers might also need professional guidance to deal with prognostic uncertainty, as they do not know what to expect and how to anticipate future decline; one of our participants referred to unpredictable disease course as a “no man’s land.”

### Integrated care

The term “no man’s land” could also be applied to the post-diagnostic care organization around people with VCI: they do not always explicitly belong in either dementia or stroke care. Although fragmented care has previously been reported within these pathways,^7,10^ our study adds that collaboration and communication between these pathways is even more limited, leading to duplication of work and thereby inefficiency and ineffective communication. Participants called for adaptations and collaborations best summarized as “Integrated care” (theme 3). In dementia literature, authors have described integrated care as close collaboration and coordination between healthcare professionals in a dementia care network.^20,48^ Suggestions for more integration in our study included closer collaboration and more communication between generalists, in primary care, and medical specialists. Medical specialists were keen to be available for advice in the post-diagnostic phase. This may arise through warm hand-offs, telephone consultation between professionals, periodic multidisciplinary meetings, or outpatient clinics by medical specialists in primary care health centers.

People with VCI and caregivers might benefit from an even wider interpretation of integrated care, further integrating post-diagnostic dementia care and stroke/rehabilitation care. Our participants stated that people with VCI could potentially fall of the radar and follow-up of stroke is often short-term or lacking, echoing literature about the neglect of cognitive consequences after stroke.^13,14^ Moreover, a Dutch study has reported that healthcare professional often do not initiate cognitive screening after stroke, although this is recommended in national guidelines.^49^ Several authors have suggested integrating rehabilitation approaches in standard dementia care.^50,51^ For people with VCI and caregivers, (cognitive) rehabilitation could be beneficial in several stages, for example after stroke, but also in the post-acute or chronic phase.^13^

### Implications for policy and practice

This study has thoroughly described the perceptions and challenges of healthcare professionals regarding post-diagnostic care for people with VCI. Most importantly, this study could be regarded as a reminder to healthcare professionals working in the dementia and stroke fields to be aware of VCI, and gather sufficient knowledge about the condition. The descriptions on how to handle specific symptoms such as apathy and fluctuations in functioning could inspire healthcare professionals to make some adjustments in their work and acknowledge the hardships of informal caregivers. Furthermore, this study stresses the importance of ‘knowing the field’, in terms of what care is provided across care pathways for people with VCI and knowing key professionals working in these pathways. Several recommendations on how to improve collaboration have been provided above.

This study could also be a starting point for formulating specific recommendations for care for the VCI group. VCI is currently not differentiated within dementia care guidelines,^52^ nor is cognitive impairment elaborated upon in general stroke guidelines.^53^ As recent guidelines on post-stroke cognitive impairment suggest, high-quality evidence to support specific recommendations is currently lacking.^54^ Therefore, studies like these can guide the field to the most important themes for professionals in providing tailored care for VCI.

### Strengths and limitations

When interpreting the results, several strengths, limitations and considerations to the design of the study should be taken into account. A major strength of this study is the diversity of the included healthcare professionals, giving us a broad understanding of the topic. Still, the psychiatric and social domain were relatively underrepresented. In addition, it is possible that participants had different perspectives on a “person with VCI”. To mitigate this limitation, we defined VCI before each focus group session. Our study was set within Dutch healthcare, which has some unique characteristics such a specialist in primary care of older people, the elderly care physician,^24^ and the national availability of case management in dementia care. However, international literature suggests structures of post-diagnostic care for stroke and dementia exist globally. Furthermore, we believe our main themes and their implications are universal and mostly independent of the international differences in organization of stroke and dementia care. In addition, we acknowledge that “specific” characteristics mentioned by the participants, such as apathy, are neither unique to VCI as compared to other causes of cognitive impairment or dementia, nor present in all people with VCI. Still, these characteristics are common in VCI and were repeatedly mentioned, emphasizing their importance to the participants.

### Conclusions

To our knowledge, this is the first study to explore healthcare professional’s perspectives on post-diagnostic care for people with VCI. Moreover, we used an integrated approach in including healthcare professionals from primary and secondary settings, working in stroke and dementia care; a first step in “breaking down the barriers” between different settings and promoting further transmural collaboration. Further research is needed to assess the appropriateness and effectiveness of the recommendations of our participants for specific and integrated care. Overall, our results call for more education of healthcare professionals about the specific challenges of VCI, in order to become more aware and help people with VCI and their caregivers step out of the “no man’s land”.

## AUTHOR CONTRIBUTIONS

Sara van de Schraaf (Conceptualization; Data curation; Formal analysis; Funding acquisition; Writing – original draft); Hanneke Rhodius-Meester (Conceptualization; Funding acquisition; Supervision; Writing – review & editing); Lindsey Rijnsent (Data curation; Formal analysis; Writing – original draft); Meyrina Natawidjaja (Data curation; Formal analysis; Writing – original draft); Esther van den Berg (Conceptualization; Writing – review & editing); Frank Wolters (Conceptualization; Writing – review & editing); Anne Visser-Meily (Conceptualization; Writing – review & editing); Geert Jan Biessels (Conceptualization; Writing – review & editing); Marjolein de Vugt (Conceptualization; Writing – review & editing); Majon Muller (Conceptualization; Supervision; Writing – review & editing); Cees Hertogh (Conceptualization; Supervision; Writing – review & editing); Eefje Sizoo (Conceptualization; Data curation; Funding acquisition; Supervision; Writing – review & editing).

## Supplementary Material

Supplementary Material

## Data Availability

The data (transcripts of focus groups, in Dutch) supporting the findings of this study are available on reasonable request from the corresponding author. The data are not publicly available due to privacy and ethical restrictions.
