# Supplementary material for: Healthcare Professionals’ Perspectives on Post-Diagnostic Care for People with Vascular Cognitive Impairment: When Help Is Needed in a “No-Man’s Land”
Source: J Alzheimers Dis. 2024 Sep 24;101(3):1001–13. doi: 10.3233/JAD-240526 (PMC11492012; doi:10.3233/JAD-240526)
Supplement: Supplementary Material [file jad-101-jad240526-s001.pdf]

Supplementary Material

Healthcare Professionals' Perspectives on Post-Diagnostic Care for People with Vascular Cognitive Impairment: When Help Is Needed in a "No-Man's Land"

Supplementary Table 1. Consolidated criteria for reporting qualitative studies (COREQ) checklist

No. Item	Guide questions/description	Details reported here and/or in the main text of the paper (page number)
**Domain 1: Research team and reflexivity**		
Personal characteristics		
1. Interviewer/ facilitator	Which author/s conducted the interview or focus group?	SvdS & ES (p. 6)
2. Credentials	What were the researcher's credentials? e.g., PhD, MD	SdS: MSc<br>LR, MN: MD<br>EB, MV: PhD<br>HR, FW, JVM, GJB, MM, CM, ES: MD PhD (Title page)
3. Occupation	What was their occupation at the time of the study?	Researcher: all<br>Psychologist: SvdS; EB, MdV<br>Geriatrician: HR, MM<br>(Junior) medical doctor: LR, MN<br>Rehabilitation physician: JVM<br>Neurologist: GJB<br>Elderly care physician: CM, ES
4. Gender	Was the researcher male or female?	SvdS, HR, LM, MN, EB, JVM, MdV, MM, ES: female<br>FW, GJB, CM: male
5. Experience and training	What experience or training did the researcher have?	SvdS completed a basic and advanced qualitative research course and has conducted a qualitative study before. ES has ample experience with qualitative analysis and moderation. LR and MN were trained by SvdS and ES about coding and analysis. MdV and CM are experts in the field of qualitative research in older people with dementia.
Relationship with participants		
6. Relationship established	Was a relationship established prior to study commencement?	Participants were approached within the professional networks of the authors through e-mail and phone. We also asked potential participants to consult their network for interested colleagues (p. 5) Therefore, some of the participants were known to the moderator and/or the observer.
7. Participant knowledge of the interviewer	What did the participants know about the researcher? e.g., personal goals, reasons for doing the research	The participants received an information leaflet with generic information about the study. Also, an introduction about the study aim and professional background of the researcher at the start of the focus groups (Supplementary Table 1).
8. Interviewer characteristics	What characteristics were reported about the interviewer/ facilitator? e.g., bias, assumptions, reasons and interests in the research topic	SvdS is a PhD-student on optimal care for people with VCI and a neuropsychologist working with older people with cognitive complaints. ES is a senior researcher supervising the work of SvdS and

		has worked as an elderly care physician with older people with dementia and stroke. Therefore, both have their own professional and research experience with the topic, which might have affected bias in the questions asked to the participants.
**Domain 2: Study design**		
Theoretical framework		
9. Methodological orientation and theory	What methodological orientation was stated to underpin the study? e.g., grounded theory, discourse analysis, ethnography, phenomenology, content analysis	The data was analysed using the inductive thematic analysis approach by Braun and Clarke (2006). (p. 7)
Participant selection		
10. Sampling	How were participants selected? e.g., purposive, convenience, consecutive, snowball.	The sampling procedure was a combination of purposeful and convenience and purposeful sampling. (p. 5)
11. Method of approach	How were participants approached? e.g., face-to-face, telephone, mail, email	Participants were approached within the professional networks of the authors through e-mail and phone. We also asked potential participants to consult their network for interested colleagues (p. 5)
12. Sample size	How many participants were in the study?	Forty participants (p. 8)
13. Non-participation	How many people refused to participate or dropped out? Reasons?	As well as personally approaching the network of the authors. We asked potential participants to consult their network for interested colleagues, therefore numbers of non-participation cannot be provided.
Setting		
14. Setting of data collection	Where was the data collected? e.g., home, clinic, workplace	Focus groups were organized at the workplace of (part of) the participants, e.g., at a general practice or a hospital. One focus group (number 6) was organised online because a live gathering of the participants was not logistically possible.
15. Presence of non-participants	Was anyone else present besides the participants and researchers?	No
16. Description of sample	What are the important characteristics of the sample? *e.g., demographic data, date*	Reported in Table 1 and start of results section (p. 8). Data was gathered between July 2022 and March 2023 (p. 6).
Data collection		
17. Interview guide	Were questions, prompts, guides provided by the authors? Was it pilot tested?	A topic list was constructed by the research team before data-collection (Supplementary Table 1, including revisions). The topic list included open-ended question focusing on the participants' views on (1) current dementia, stroke and VCI post-diagnostic care and support (2) possible caveats in post-diagnostic care and support for people with VCI and caregivers and (3) how to organize this care according in terms of collaboration and key stakeholders. The topic list was revised several times during data collection, primarily to accommodate the different participants or settings. (p. 6) There was no pilot test because of the focus group design.

18. Repeat interviews	Were repeat interviews carried out? If yes, how many?	N.A.
19. Audio/visual recording	Did the research use audio or visual recording to collect the data?	All focus groups were audiotaped and transcribed verbatim (p. 6)
20. Field notes	Were field notes made during and/or after the interview or focus group?	Field notes were taken during each focus group session, mainly focusing on non-verbal communication. After each focus group session, we debriefed according to a checklist and noted impressions. (p. 6)
21. Duration	What was the duration of the interviews or focus group?	Focus groups lasted a maximum of 90 minutes (p. 6)
22. Data saturation	Was data saturation discussed?	Data-collection ended at thematic saturation, meaning additional information and data does not contribute to new (sub-)themes. At the seventh focus group, no novel topics were discussed that led to different (sub-) themes; therefore, we concluded that saturation was reached. (p. 6)
23. Transcripts returned	Were transcripts returned to participants for comment and/or correction?	No. (p. 6)
**Domain 3: Analysis and findings**		
Data analysis		
24. Number of data coders	How many data coders coded the data?	At least two researchers (SvdS and LR or MN, trained by SvdS and ES) independently coded each transcript (p. 7)
25. Description of the coding tree	Did authors provide a description of the coding tree?	Supplementary Figure 1
26. Derivation of themes	Were themes identified in advance or derived from the data?	An inductive approach was used: i.e. themes were derived from the data (p. 67)
27. Software	What software, if applicable, was used to manage the data?	MAXQDA 2022 (p. 7)
28. Participant checking	Did participants provide feedback on the findings?	After data-collection and analysis, we organized two opportunities for our participants to attend a presentation of the outcomes and proposed themes. […] Participants were asked to comment on whether the proposed themes reflected their understanding of the topic, that is an interpretive stance on member checking (assessing the trustworthiness of the analysis of the data). (p. 6-7)
Reporting		
29. Quotations presented	Were participant quotations presented to illustrate the themes/findings? Was each quotation identified? e.g., participant number	Reported in the Results section. (p. 8-16)
30. Data and findings consistent	Was there consistency between the data presented and the findings?	Reported in the Results section. (p. 8-16)
31. Clarity of major themes	Were major themes clearly presented in the findings?	Reported in the Results section. (p. 8-16)
32. Clarity of minor themes	Is there a description of diverse cases or discussion of minor themes?	Reported in the Results section. (p. 8-16)

Supplementary Table 2. Topic list of the focus groups (final version 5) with change log [*between brackets]*

Time (total 90 min)	Activity	Details/questions asked:
10 min	Welcome, house rules & introduction round.	- Moderator: Opens meeting, greets everyone. Introduces themselves and observer.<br>- Observer: Introduces themselves. Gives a two-sentence introduction of the study.<br>- Moderator: discusses some house rules:<br>- Informally addressing each other.<br>- Goal is to create a discussion. However, please allow others to finish their sentence (also because of the quality of the recordings).<br>- Talking through the moderator is not necessary, please react to each other.<br>- Focus group will be recorded and transcribed while omitting identifiable information.<br>- Moderator: asks participants to introduce themselves (name and occupation)
5 min	Further introduction of the study.	- Moderator: introduces the observer.<br>- Observer: gives substantive introduction of the study:<br>→ definition Vascular Cognitive Impairment (VCI) in *this* study: people with cognitive impairment, ranging from mild levels of cognitive impairment to dementia level, where vascular aetiology is the most likely and prominent cause.<br>- Observer: defining "care": study focusing on post-diagnostic care and support, not the (technicalities of) the diagnosis of VCI.
55 min	A. Opening question + topic list.<br>Structure:<br>1. Main topics<br>o Optional follow-up questions	- Moderator asks opening question:<br>**What comes to mind when thinking about someone with cognitive complaints due to vascular aetiology?**<br>1. What are the needs of this group, according to you?<br>o And what are the needs of their caregivers? *[removed in version 5]**<br>2. How is care for people with VCI delivered in your work setting?<br>o How do you see your own role in the care for people with cognitive disorders? *[added in version 4]*<br>i. What do you need to fulfil this role?<br>o What care and support is currently available for people with VCI (and caregivers)?<br>o What is going well in care and support for this group?<br>o What aspects require additional focus or awareness?<br>o Which aspects or care and support are specific or appropriate for people with VCI and caregivers?<br>o Are there things you approach differently with regards to VCI as compared to other groups with cognitive disorders (such as Alzheimer's disease)?<br>o Are there differences in the care and support for the VCI group between stroke care and dementia/memory clinic care? Why? [added in version 5]<br>3. Is there enough knowledge about this group in different settings? (such as primary care?) *[added in version 4]*<br>o Would more knowledge about this group lead to better care for people with VCI and their caregivers?<br>4. How is the collaboration with other healthcare professionals in other settings (primary care/secondary care)? *[added in version 4]*<br>o What do you need from secondary care professionals to keep care and support within primary care?

		○ How is the collaboration with stroke rehabilitation? [a*dded in version 5]*<br>5. How should we optimally organise care for people with VCI?<br>○ What is needed to realize care effectively?<br>○ What should be organized differently in the ideal scenario? [added in version 5]<br>○ Are there elements from different care pathways (such as dementia and stroke) that should be transferred to other care pathways with regards to the VCI group? [added in version 5]<br>○ To which healthcare professional do you refer in which scenario? [removed in version 5]<br>○ Who are the key professionals in VCI care? [removed in version 5]
10 min	B. Take home message & prioritising.	Given there is enough time, the moderator asks all participants in order to reflect on the following questions:<br>1. What do you take home from this focus group discussion?<br>○ What do you want us [the researchers] to take home?<br>2. Of all the topics we have discussed, what are the most important aspects to you?
10 min	Conclusion of the session.	- Moderator: gives observer opportunity to ask additional questions.<br>- Moderator: asks closing question: Did you miss any topics during this discussion? Are there any burning remarks?<br>- Moderator: concluding remarks, asks the participants about their general experience with the focus group.<br>Observer: briefly discussing future developments of the study and thanks the participants for participating.
After focus group (15-30 min)	Debriefing	Moderator and observer.
OPTIONAL: if A+B do not elicit enough discussion†	C. Presenting previous study.	-Moderator introduces observer<br>-Observer: presents themes from interview study with people with VCI & caregivers about care needs.
OPTIONAL†	C. Response of participants to previous study.	1. What do you think of these themes, can you react to them?<br>2. With the previous discussion (part A) in mind, do these findings elicit new thoughts or complementary information? Why?<br>3. Which care needs stand out to you?<br>4. What types of care and support could fulfil those needs?<br>5. How could we organize this?

*Changes in later versions of the topic list could be summarized in the following reasons.

1. Elements were removed when topics (a) were already widely discussed in previous focus groups (saturation) or (b) would arise spontaneously in every discussion (e.g., the matter of care and support for caregivers of people with VCI).
2. Elements were added when topics (a) would come up frequently, but thick descriptions were not yet achieved or (b) specific questions were needed to address professionals in another settings (e.g., primary care as opposed to hospital settings).

†This element was changed to optional after the first focus group [version 2] as it could potentially induce bias in the responses of the participants. In the end, this optional element was not used in any of the focus groups, because the discussions were lengthy and substantive enough with only elements A&B.

Supplementary Figure 1. Coding cloud themes of the perspectives of healthcare professionals on post-diagnostic care for people with VCI.

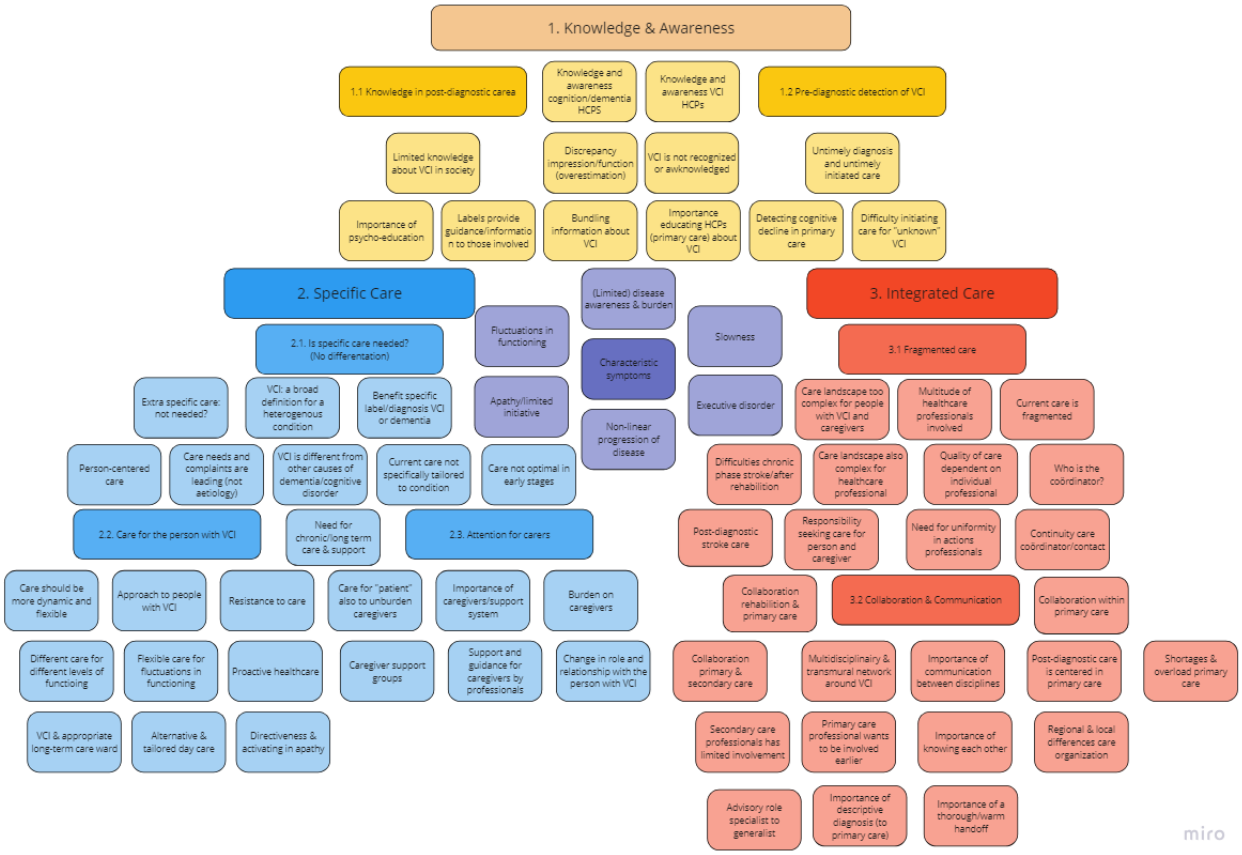

Coding cloud was created on miro.com.
Characteristic symptoms (in purple) are displayed in the middle of the cloud. Although these codes do not represent perspectives (and therefore are not a theme in the data), they were mentioned repeatedly by the participants in all major themes and thus displayed in this figure.
